# Efficient Solar Light Photocatalyst Made of Ag_3_PO_4_ Coated TiO_2_-SiO_2_ Microspheres

**DOI:** 10.3390/nano13030588

**Published:** 2023-02-01

**Authors:** Sudipto Pal, Sanosh Kunjalukkal Padmanabhan, Amruth Kaitheri, Mauro Epifani, Antonio Licciulli

**Affiliations:** 1 Department of Engineering for Innovation, University of Salento, Via Monteroni, 73100 Lecce, Italy; 2 Istituto per la Microelettronica e Microsistemi, IMM-CNR, Via Monteroni, 73100 Lecce, Italy; 3 Institute of Nanotechnology, CNR Nanotec, Consiglio Nazionale Delle Ricerche, Via Monteroni, 73100 Lecce, Italy

**Keywords:** silver phosphate, titania–silica microsphere composite, solar photocatalyst

## Abstract

Solar light active photocatalyst was prepared as silver phosphate (Ag_3_PO_4_) coating on titania–silica (TiO_2_–SiO_2_) microspheres. Titania–silica microsphere was obtained by spray drying TiO_2_–SiO_2_ colloidal solutions, whereas Ag_3_PO_4_ was applied by wet impregnation. XRD on the granules and SEM analysis show that the silver phosphate particles cover the surface of the titania–silica microspheres, and UV-visible diffuse reflectance analysis highlights that Ag_3_PO_4_/TiO_2_–SiO_2_ composites can absorb the entire visible light spectrum. BET measurements show higher specific surface area of the composite samples compared to bare Ag_3_PO_4_. Photocatalytic activity was evaluated by dye degradation tests under solar light irradiation. The prepared catalysts follow a pseudo-first-order rate law for dye degradation tests under solar light irradiation. The composite catalysts with an Ag_3_PO_4_/TiO_2_–SiO_2_ ratio of 1:1.6 wt% show better catalytic activity towards both rhodamine B and methylene blue degradation and compared with the results with uncoated TiO_2_–SiO_2_ microspheres and the benchmark commercial TiO_2_ (Evonik-P25) as a reference. The composite photocatalyst showed exceptional efficiency compared to its pristine counterparts and reference material. This is explained as having a higher surface area with optimum light absorption capacity.

## 1. Introduction

Purification of polluted surfaces, air, and water is pivotal for maintaining a healthy environment [1,2]. Photocatalysis can be considered a promising solution for various purification applications [3,4,5]. Many attempts have been made to produce visible light active photocatalysts for polluted air and water treatment [6,7,8,9,10,11,12]. Semiconductor photocatalysts are a good choice since they are highly efficient, eco-friendly, and chemically stable [13,14,15]. Titanium dioxide (TiO_2_) is one of the most studied photocatalysts with diverse applications, owing to its excellent physical and chemical properties with higher photo and chemical stability [16,17,18,19,20]. Because of its wide band gap, TiO_2_ catalyst is photo-active only under UV light exposure that limits its applications [21,22,23,24]. To utilize sunlight as the radiation source, it is necessary to make the TiO_2_ visible light active, which also extends its use in indoor conditions where UV light is filtered by glass and LED lights are used as the light source. To make it visible light active several efforts have been reported. The most common method is to couple with noble metal nanoparticles (Au, Ag, and Cu) [22,23,24], metal oxides (CuO/Cu_2_O, CeO_2_, Fe_2_O_3_, WO_3_ etc.), and nitrides like g-C_3_N_4_ [25,26,27,28].

The significant photo-oxidative characteristics of Ag_3_PO_4_ for producing oxygen from water and the destruction of organic contaminants under visible light irradiation make it a very promising photocatalyst [29,30,31,32,33]. In addition, silver orthophosphate possesses a lower band gap energy (2.45 eV) compared to titanium dioxide (3.2 eV) and therefore can be activated in the visible region of the electromagnetic spectrum, where TiO_2_ is idle. Despite the above-mentioned outstanding qualities; its size, cost of preparation, poor solubility in water, reduction of silver ions (Ag^+^) to metallic silver (Ag^0^) during the photocatalytic process (referred as photo-corrosion), and lower specific surface area (SSA) are some of the limitations of this compound [21,34,35]. Ag_3_PO_4_ alone as a photocatalyst also suffers from lower efficiency due to the fast recombination rate of the photo-generated charge carriers [36,37,38]. These drawbacks can be minimized by creating multiphase materials that include Ag_3_PO_4_ with semiconductors like TiO_2_ and carbon compounds like graphene, graphitic carbon nitride, and graphene oxide, etc. [34,39,40].

Silica supported titania could serve as an ideal substrate due to its higher thermal stability and enhanced mechanical properties, whereas TiO_2_–SiO_2_ microspheres could have the advantage of reusability due to its first precipitation and separation from the treated media [15,41]. Moreover, TiO_2_–SiO_2_ microspheres can reach high surface area and porosity, uniform shape, greater dispersion, and low cost of production [15]. As a result, they are considered to be a great carrier material. This type of material can cut the costs of the bulk silver phosphate photocatalysts as well as improve the photocatalytic activity. Additionally, the high adsorption capability of amorphous silica makes it useful in preparing supported catalysts.

In this work, efficient titania–silica microspheres/Ag_3_PO_4_ catalysts were prepared. The chemical composition, shape, and optical characteristics of the synthesized silica titania microspheres/Ag_3_PO_4_ catalysts were studied in detail. Photo degradation of rhodamine B (RhB) and methylene blue (MB) dyes as the model pollutant in water under solar radiation was used to assess the photocatalytic efficacy of the synthesized catalysts.

## 2. Materials and Methods

### 2.1. Experimental Section

The silver orthophosphate photocatalysts were synthesized at room temperature using a co-precipitation technique, whereas titania–silica microspheres were prepared using a spray drying technique. The mesoporous TiO_2_–SiO_2_ microspheres (composition: 80 wt% TiO_2_/20 wt% SiO_2_, hence denoted as TS82) were prepared following a modified procedure described in our previous works [15,42]. A nonionic copolymer surfactant, Pluronic F127 ([C_3_H_6_O·C_2_H_4_O]_x_, Powder, BioReagent, Sigma-Aldrich, St. Luis, MO, USA) was used as the mesopore generating agent whereas titanium tetraisopropoxide (Ti(O^i^Pr)_4_, 97%, Sigma-Aldrich) and tetraethoxysilane (Si(OC_2_H_5_)_4_, 97%, Sigma-Aldrich) were used as the inorganic precursors for TiO_2_ and SiO_2_. A spray drying method was used to prepare the TiO_2_–SiO_2_ microspheres. Briefly, an aqueous suspension of TiO_2_-SiO_2_ (TiO_2_/SiO_2_ weight ratio of 80/20) was prepared by hydrolysis-condensation of Ti(O^i^Pr)_4_ and Si(OC_2_H_5_)_4_ in acidic pH, which was used as the stock solution for spray drying. Then the suspension was spray dried with a high pressure atomizer (ICF Welko, commercial grade, I.C.F. & Welko S.P.A., Maranello, Italy) that resulted the production of the TS82 microspheres. Then the microspheres were calcined at 550 °C for 6 h with the heating and cooling rate of 1 °C/min to remove the organic content and enhance the crystallization of TiO_2_ as well. For silver phosphate coating, silver nitrate (AgNO_3_, Crystal, BAKER ANALYZED™ A.C.S. Reagent, J.T. Baker™, Fisher Scientific Co. L.L.C., Pittsburgh, PA 15275, USA) and di-potassium hydrogen phosphate (ACS Reagent ≥ 98%, Sigma-Aldrich) were used as the precursors. Three compositions with different TS82: Ag_3_PO_4_ ratios were prepared. The weight ratios of TS82 to silver phosphate that were investigated throughout the work were 1:0.42 (TS82-AgP4), 1:0.84 (TS82-AgP8), and 1:1.68 (TS82-AgP16). TS82 powder (50 mg) was first dispersed in 100 mL water under vigorous stirring, and then the required amount of silver nitrate aqueous solution was added and stirred overnight to let the Ag^+^ ions be adsorbed on the surface of the TS82 microspheres. Dropwise addition of an aqueous dipotassium hydrogen phosphate (K_2_HPO_4_·3H_2_O) solution to the silver deposited TS82 suspension followed, and a yellow precipitate formed. The precipitate was centrifuged, washed several times with deionized water and ethanol, and dried at 70 °C overnight to give dried composite powder samples. In a similar approach, pure Ag_3_PO_4_ was produced by keeping the Ag/P molar ratio at 3:1. To identify the role of TiO_2_ in photocatalysis, pure silica microspheres/Ag_3_PO_4_ composite was also prepared following the similar method described above. Aeroxide P25 titanium dioxide nanopowder (Evonik Operations GmbH, Essen, Germany) was used as the benchmark photocatalyst for comparison.

### 2.2. Characterization

The X-ray diffraction (XRD) patterns were recorded on a Rigaku Ultima diffractometer (Rigaku Corporation, Akishima-shi, Tokyo, Japan) with Cu Kα radiations generated at 40 kV and 20 mA. The nitrogen adsorption/ desorption measurements were performed using NOVA 2000e (Quantachrome, Anton Paar USA Inc., Timber Ridge, VA, USA) apparatus at a temperature of −196 °C. The samples were degassed for 3 h at 105 °C. The multipoint Brunauer–Emmett–Teller (BET) technique was used to calculate the specific surface area (SSA) using adsorption data in the relative pressure range of 0.05–0.35. The UV-visible absorption spectra and diffuse reflectance spectra were measured using an Agilent Carry 5000 UV-visible-NIR spectrophotometer (Agilent Technologies Inc., Santa Clara, CA, USA) in the wavelength range of 200 to 800 nm equipped with a standard PTFE 150 mm diameter integrating sphere. Fourier transform infrared spectra (FTIR) measurements were carried out using a Nicolet 6700 spectrometer (Thermo Fisher Scientific Inc., Waltham, MA, USA) in a diffuse reflectance setup, after dispersing the sample powders in KBr and accumulating 36 scans over 4000–400 cm^−1^ wavenumber range. Microstructural investigation was performed with an EVO-Zeiss (Carl Zeiss Microscopy GmbH, Jena, Germany) field emission scanning electron microscope (FESEM).

### 2.3. Evaluation of Photocatalytic Activity

Photocatalytic activity was evaluated using dye degradation tests using organic Rhodamine B (RhB) and Methylene Blue (MB) as the model pollutants. The experiments took place in a photocatalytic reactor that includes a solar simulator lamp, magnetic stirring system, and cooling apparatus. The simulation of the spectrum of natural sunlight was achieved using a 300 W tungsten lamp (Sanolux, Radium Lampenwerk, Irradiance 41.4 W m^−2^ at 380–780 nm; 13.6 W m^−2^ at 315–400 nm and 3.0 W m^−2^ at 280–315 nm wavelength. The photocatalysts (1 g L^−1^) were dispersed in 200 mL of RhB/MB aqueous solution (10 ppm) and the suspension was irradiated with solar light. The suspensions were stirred in the dark overnight before exposition to solar light to achieve the adsorption–desorption equilibrium. The photocatalytic degradation of RhB and MB was monitored by measuring the intensity of the dyes’ absorption bands at 554 and 650 nm, respectively. An Agilent Cary 5000 series UV-visible spectrophotometer was used to record the absorption spectra of the irradiated solution. The solution was tested at certain time intervals and separated from the catalysts by centrifugation before the spectrum measurement.

## 3. Results and Discussion

**Figure 1:**
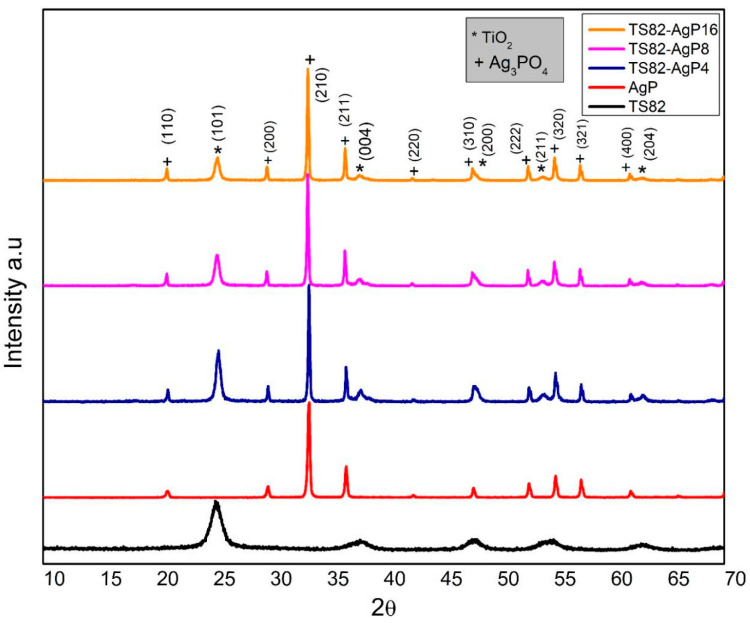
XRD patterns of TS82, pure Ag_3_PO_4_ (AgP), and composites with different TS82/AgP ratios.

Figure 1 shows the XRD pattern of TS82, AgP, and TS82-AgP composites with different TS82: AgP ratios. XRD patterns of TS82 show monophasic anatase crystalline phase corresponding to crystalline peaks at 2θ values of 25.38°, 37.04°, 48.12°, 54.36°, and 62.7° assigned to (101), (004), (200), (211), and (204) crystalline planes of the anatase TiO_2_ (JCPDS no. 84-1286). The diffraction pattern of the TS82 microspheres does not show any evident peak corresponding to silica, as it forms an amorphous phase. The indexed diffraction peaks of pure silver phosphate (AgP) show the formation of body-centered cubic silver phosphate (JCPDS No. 84-0512). The crystalline peaks of silver phosphate at 2θ values of 20.93°, 29.88°, 33.31°, 36.69°, 42.65°, 47.94°, 52.73°, 55.15°, 57.35°, and 61.79° assigned to (110), (200), (210), (211), (220), (310), (222), (320), (321), and (400) crystalline planes respectively. All the synthesized TS82/AgP composite catalysts show peaks ascribed to both AgP and anatase titania nanocrystalline phases, confirming the formation of TS82/AgP composites without any intermediate phase.

**Figure 2:**
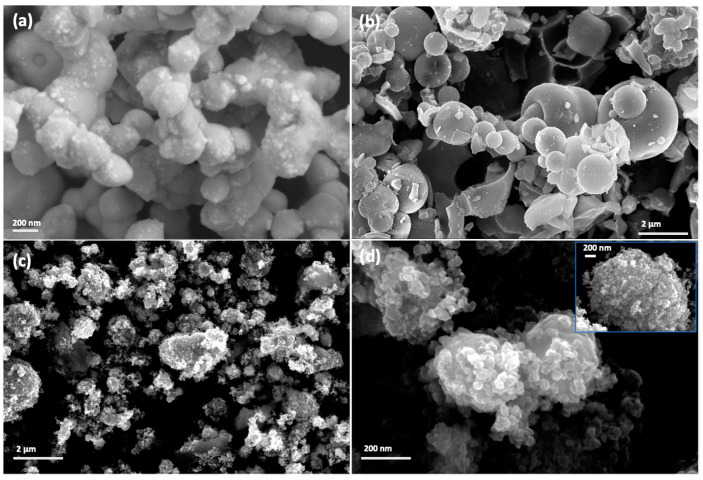
SEM images of (**a**) Ag_3_PO_4_, (**b**) TS82, (**c**) TS82-AgP16 and (**d**) higher magnification image of TS82-AgP16.

The morphology of Ag_3_PO_4_ and TS82 microspheres is presented with the SEM micrograph (Figure 2a,b, respectively). The Ag_3_PO_4_ particles have an irregular and polyhedral morphology and exist in an aggregated state. From Figure 2b, it is clear that TS82 microparticles possess spherical morphology and smooth surfaces. Some spheres are in a broken state, unveiling the hollow nature of the microspheres. The size of the TS82 could be estimated in the range of submicron to 5 μm. The SEM micrograph of the composite catalyst TS82/AgP16 in Figure 2c shows that the Ag_3_PO_4_ nanoparticles are uniformly distributed on the surface of TS82 microspheres and titania silica microspheres are decorated with Ag_3_PO_4_ nanoparticles with an average size of 40 nm (inset of Figure 2d). It is also noteworthy to observe that in case of pristine Ag_3_PO_4_, aggregated particles of 200 to 300 nm were formed due to rapid nucleation in the reaction solution leading to crystal growth [43]. However, this feature is absent in the composite material indicating that the TS82 microspheres act as nucleation controlling medium leading to nearly uniform distribution of the small sized (~50 nm) Ag_3_PO_4_ nanoparticles. Initial adsorption of the Ag^+^ ions on negatively charged TiO_2_–SiO_2_ surfaces could promote the limited nucleation of the Ag_3_PO_4_ nanoparticles.

The SSA of the samples obtained from N_2_ adsorption isotherm by BET measurements are reported in Table 1. It is evident that the optimum amount of silica addition to TiO_2_ nanostructure results in the formation of mesoporous structures showing higher SSA that enhance the overall photocatalytic efficiency [41,42] In this work, the highest SSA was found for the TS82 microspheres (173 m^2^/g), which was used as a support catalyst of Ag_3_PO_4_ which has a very low SSA of 4.1 m^2^/g. The composites TS82-AgP4, TS82-AgP8, and TS82-AgP16 showed a surface area of 114, 109 and 98 m^2^/g, respectively. For the composite catalysts, a decrease in surface area with the increase in AgP amount can be observed, which is in agreement with the individual SSA of TS82 and Ag_3_PO_4_.

**Figure 3:**
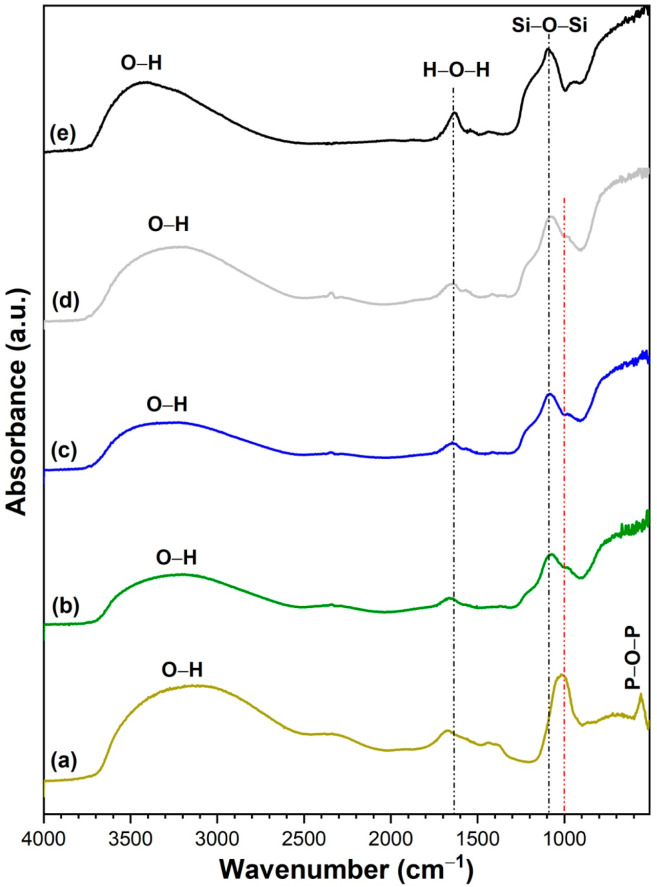
FTIR spectra of (**a**) Ag_3_PO_4_, (**b**) TS82-AgP4, (**c**) TS82-AgP8, (**d**) TS82-AgP16, and (**e**) TS82 microspheres.

Figure 3 shows the FTIR spectra of the composite samples (TS82-AgP4, TS82-AgP8, and TS82-AgP16) along with their pristine counterpart (AgP and TS82). All the samples show a broad vibrational band centered at 3400 cm^−1^ that corresponds to O–H stretching vibration, while the peak at 1657 cm^−1^ corresponds to the bending vibration of the adsorbed water molecules [21]. Pure Ag_3_PO_4_ shows strong vibrations centered around 1000 cm^−1^ and 547 cm^−1^ that arise from P–O asymmetric stretching vibrations and P–O–P bending vibrations arising from ${\mathrm{PO}}_{4}^{3–}$ group of silver phosphate [21,44]. On the other hand, TS82 shows distinct vibration bands centered at 1092 cm^−1^ due to Si–O–Si asymmetric and symmetric stretching vibrations of the silica network [42,45]. With the increasing amount of Ag_3_PO_4_ in the composite, the peak intensity of silica decreases, while that for phosphates increases, indicating the formation of TS82-AgP composites.

**Figure 4:**
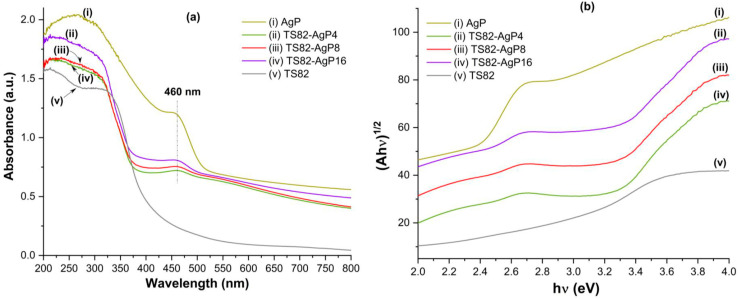
(**a**) Optical absorption spectra of different samples and (**b**) Plot of (Ah*v*)^1/2^ against the incident photon energy (eV) calculated from the absorption spectra plotted in (**a**).

The optical diffuse reflectance absorption spectra of different samples are shown in Figure 4a. All the spectra were recorded in the UV-visible range to determine the light absorption capacity and the optical band gap of each composition. The detailed calculation procedure is described elsewhere [21]. TS82 sample shows strong absorption in the UV region whereas there is no absorption in the visible region, indicating its inactivity in the visible region. The pure Ag_3_PO_4_ sample shows strong absorption starting from UV to the visible wavelength range centered at 460 nm and broad absorption in the 500–800 wavelength range. With the deposition of Ag_3_PO_4_ on TS82 microspheres, strong absorption in the visible region (400–800 nm) is observed with the absorption peak at 460 nm. With increasing loading of Ag_3_PO_4_, the intensity of the absorption peak also increases, indicating higher visible light absorption with increasing Ag_3_PO_4_ content. This is further confirmed from the band gap energy measurements that are reported in Table 1 derived from Figure 4b.

**Table 1:** BET surface area and band gap determination of different composites.

Samples	BET Surface Area (m^2^/g)	Band Gap Energy (eV) ^1^
TS82	173	3.18
AgP	4.1	2.41
TS82-AgP4	114	2.52
TS82-AgP8	109	2.48
TS82-AgP16	98	2.45

^1^ Obtained from Figure 4b.

**Figure 5:**
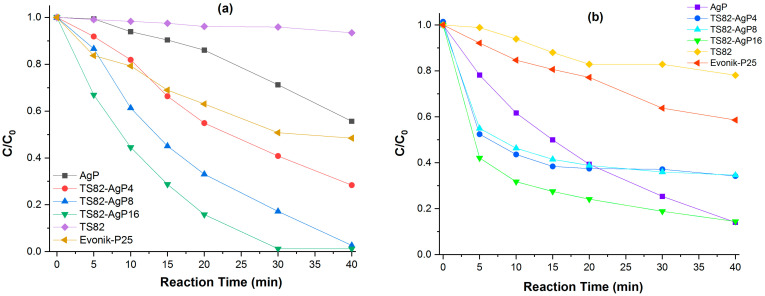
Plot of C/C_0_ versus reaction time (solar light irradiation time) of different photocatalyst samples performed with (**a**) RhB and (**b**) MB dye solutions.

The photocatalytic activity of TS82-AgP samples has been studied using dye degradation techniques in the presence of solar radiation. The spectral evolution of the absorption band of RhB and MB in the presence of different photocatalyst is presented in Figures S1 and S2. A significant amount of adsorption was observed in the case of composite samples (more prominent for MB). The C/C_0_ vs. reaction time with RhB and MB dyes has been plotted where C_0_ represents the initial concentration and C represents the concentration of the dye solution with respect to the exposure duration (Figure 5). Evonik-P25 is used as a reference photocatalyst for the comparative study of the photocatalytic properties of the aforementioned dyes. The photodegradation process for both dyes followed pseudo-first-order reaction kinetics and a pseudo-first-order rate constant (K). The maximum rate constant value is found for the TS82-AgP16 composite in both cases. It is clearly evident from the graph that the composites have much better photocatalytic efficiencies than the pure TS82 and pure AgP. There is an increase in the slope of the curve for composites, which indicates the enhancement of the photocatalytic property. The photocatalytic reaction rate constant for all the samples for RhB and MB degradation is reported in Table 2. It is observed that there is a considerable increase in the rate constant of the composites as the concentration of AgP is increased and reaches its maximum for TS82-AgP16_,_ which indicates the excellent photocatalytic behavior of TS82-AgP16. We did not observe any further increase in photocatalytic efficiency by increasing the amount of Ag_3_PO_4_. The rate constant (K) of TS82-AgP16 is found to be 12.6 × 10^−2^ min^-1^ and 4.84 × 10^−2^ min^-1^ for RhB and MB degradation, respectively. The rate constants of all composites are greater than the rate constants of pure TS82 and AgP. It is assumed that, with increasing AgP content, the photocatalytic efficiency also increased due to the higher visible light absorption capacity of TS82-AgP16 (Figure 3b), but further increase results decrease in photocatalytic activity (data not shown) that might be due to lower SSA and formation of separated AgP clusters. We also investigated the photocatalytic activity of Ag_3_PO_4_ deposited on SiO_2_ microspheres prepared in a similar way to TS82 microspheres. Poor photocatalytic efficiency reported in Figure S3, Table S1 indicates that TiO_2_ in the composite samples may play a significant role in photocatalytic dye degradation.

**Figure 6:**
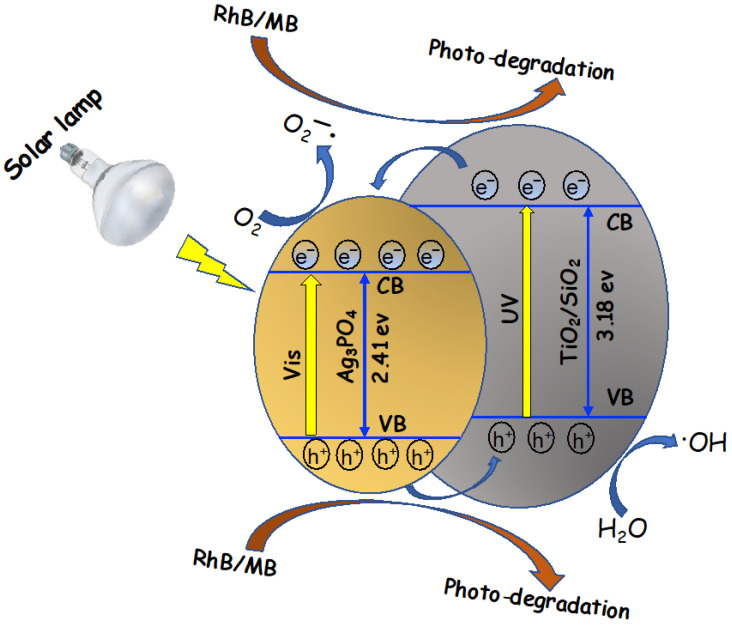
Schematic representation of the enhanced photodegradation mechanism of the Ag_3_PO_4_/TiO_2_–SiO_2_ nanocomposite under solar light irradiation.

A possible reaction pathway of the photodegradation process based on the experimental results obtained is depicted in Figure 6. It is evident that the photocatalytic mechanism of the Ag_3_PO_4_-TiO_2_ composites with different morphologies is well reported [38,46,47,48,49,50,51,52]. According to the previous reports, the alignment of the valance band (VB) and conduction band (CB) of Ag_3_PO_4_-TiO_2_ heterostructure favors the electron-hole charge separation that accelerates the photocatalytic process [46,50]. In our system, when the solar light falls on the composite photocatalyst, the electrons in VB are excited to the CB of Ag_3_PO_4_ leaving the holes in the VB. Since the VB position (+2.9 eV vs. NHE) of Ag_3_PO_4_ is lower than TiO_2_ (VB position +2.7 eV vs. NHE), the holes in the VB of Ag_3_PO_4_ can be quickly transferred to the VB of TiO_2_ (hence TiO_2_/SiO_2_) thus creating the charge separation. Mesoporous TiO_2_/SiO_2_ microstructure may also act as a hole trapping agent that inhibits the carrier recombination favoring the photocatalytic process. These valence band holes may come into direct contact with the water molecules adsorbed by the composite catalyst, resulting in the formation of hydroxyl radicals. These radicals play a major role in the degradation of RhB and MB. On the other hand, the photo-generated electrons in Ag_3_PO_4_ can migrate to the Ag_3_PO_4_ surface and interact with the adsorbed water molecules to generate oxide species such as reactive superoxide radical anions. It is unlikely that the excited electrons in CB of Ag_3_PO_4_ would transfer to the CB of TiO_2_ since CB of TiO_2_ (−0.5 eV vs. NHE) is more negative than CB of Ag_3_PO_4_ (0.45 eV vs. NHE) [47,49,51,52]. Rather, some photo-excited electrons may jump to Ag_3_PO_4_ CB due to the absorption edge of TiO_2_ at 380 nm that may arise from the irradiance of the solar lamp, which also emits low intensity UV-A and UB-B lights. This is supported by the poor photocatalytic activity of the Ag_3_PO_4_-SiO_2_ composite compared to Ag_3_PO_4_-TiO_2_/SiO_2_ (Figure S3, Table S1). The maximum activity of the TS82-AgP16 composite is attributed to an efficient charge separation mechanism and a larger surface area than pure silver phosphate.

## 4. Conclusions

Efficient tandem photocatalysts with silver phosphate deposited on titania–silica microspheres were synthesized for solar light driven photocatalytic processes. The composite catalysts proved to be very different from both the titania–silica microsphere and silver phosphate alone in many respects. The energy band gap of titania–silica microsphere from the UV region shifted to the visible range in the case of composite catalyst. To explain the better performance of the composite catalysts, we suggest that photogenerated electrons in the conduction band of silver phosphate are transported to the TS82 surface where they combine with atmospheric oxygen to create reactive superoxide radical anions, whereas holes may come into direct contact with the water molecules, resulting in the formation of hydroxyl radicals. An optimum level of AgP loading on titania–silica microspheres resulted in higher specific surface area with higher visible light absorption capacity that makes the composite catalysts much more efficient. The higher surface of the composite is converted to a higher number of reaction sites for the catalyst for the dye molecules, increased light absorption, and accelerated the photo-degradation reaction. Moreover, the micrometric size of the TS82 microspheres make it easy to collect by filtration. This supported system can be easily scaled up and eventually extended to other supports.

## Figures and Tables

**Table 2:** Photocatalytic reaction rate constants of RhB and MB dye degradation under solar light.

Photocatalysts	RhB	MB
	* *K* (10^−2^)	* *K* (10^−2^)
TS82	0.16	0.649
AgP	1.46	4.0
TS82-AgP4	3.24	2.0
TS82-AgP8	8.55	2.14
TS82-AgP16	12.6	4.84
Evonik-P25	1.84	1.35

* Reaction rate constant, min^−1^; average of three measurements of each photocatalyst.

## Data Availability

The data are available from the corresponding author upon reasonable request.

## Supplementary Materials

The following supporting information can be downloaded at: https://www.mdpi.com/article/10.3390/nano13030588/s1, Figure S1. Evolution of the UV-visible absorption spectra of RhB dye aqueous solution (10 ppm) under solar light irradiation performed with different photocatalyst samples as indicated in the figure title; Figure S2. Evolution of the UV-visible absorption spectra of MB dye aqueous solution (10 ppm) under solar light irradiation performed with different photocatalyst samples as indicated in the figure. Figure S3. Plot of C/C_0_ with reaction time performed with SiO_2_-AgP16 and TS82-AgP16 microsphere composite using (a) RhB and (b) MB dye Table S1. Photocatalytic reaction rate constant of the corresponding samples.
