# Supplementary material for: Efficient Solar Light Photocatalyst Made of Ag3PO4 Coated TiO2-SiO2 Microspheres
Source: Nanomaterials (Basel). 2023 Feb 1;13(3):588. doi: 10.3390/nano13030588 (PMC9921011; doi:10.3390/nano13030588)
Supplement: Supplementary file 1 [file nanomaterials-13-00588-s001.zip › nanomaterials-2170584-supplementary.pdf]

Supporting Information

Efficient Solar Light Photocatalyst Made of Ag_3PO_4 coated TiO_2-SiO_2 Microspheres

Sudipto Pal[1*], Sanosh Kunjalukkal Padmanabhan[1*], Amruth Kaitheri[1], Mauro Epifani[2] and Antonio Licciulli[1,3]*

1 Department of Engineering for Innovation, University of Salento, Via Arnesano, 73100 Lecce, Italy.
2 Istituto per la Microelettronica e Microsistemi, IMM-CNR, Via Monteroni, 73100 Lecce, Italy.
3 Institute of Nanotechnology, CNR Nanotec, Consiglio Nazionale Delle Ricerche, Via Monteroni, 73100 Lecce

***** Correspondence: sudipto.pal@unisalento.it; sanosh.padmanabhan@unisalento.it; antonio.licciulli@unisalento.it

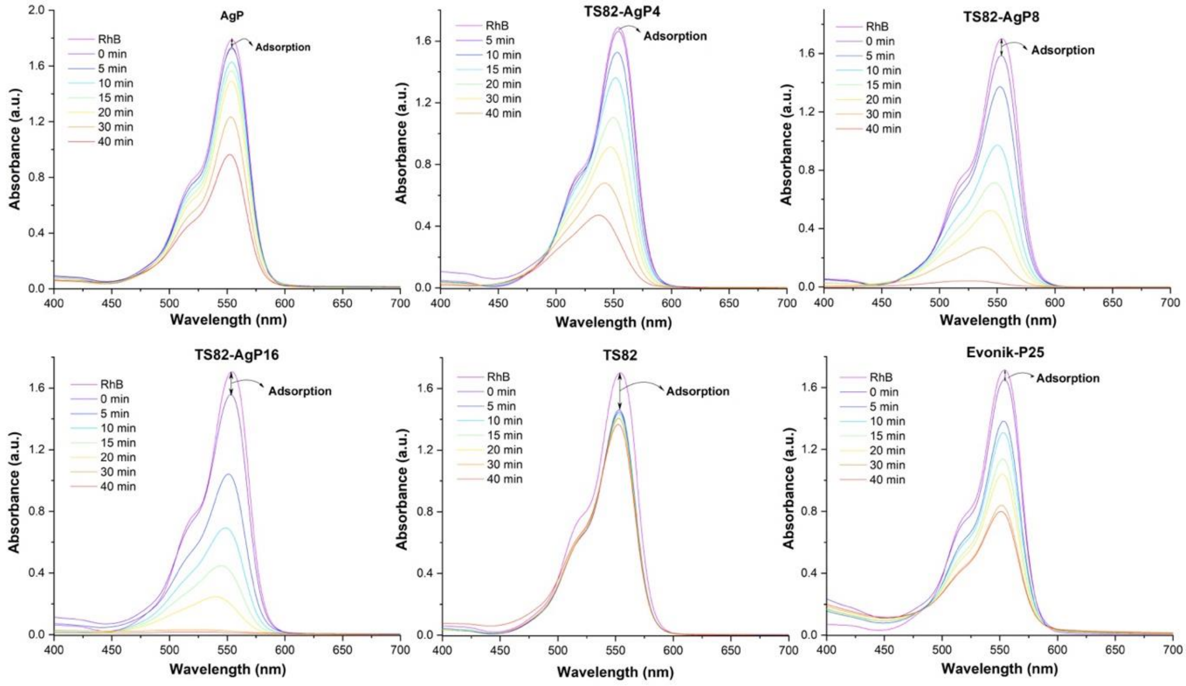

Figure S1. Evolution of the UV-Visible absorption spectra of RhB dye aqueous solution (10 ppm) under solar light irradiation performed with different photocatalyst samples as indicated in the figure.

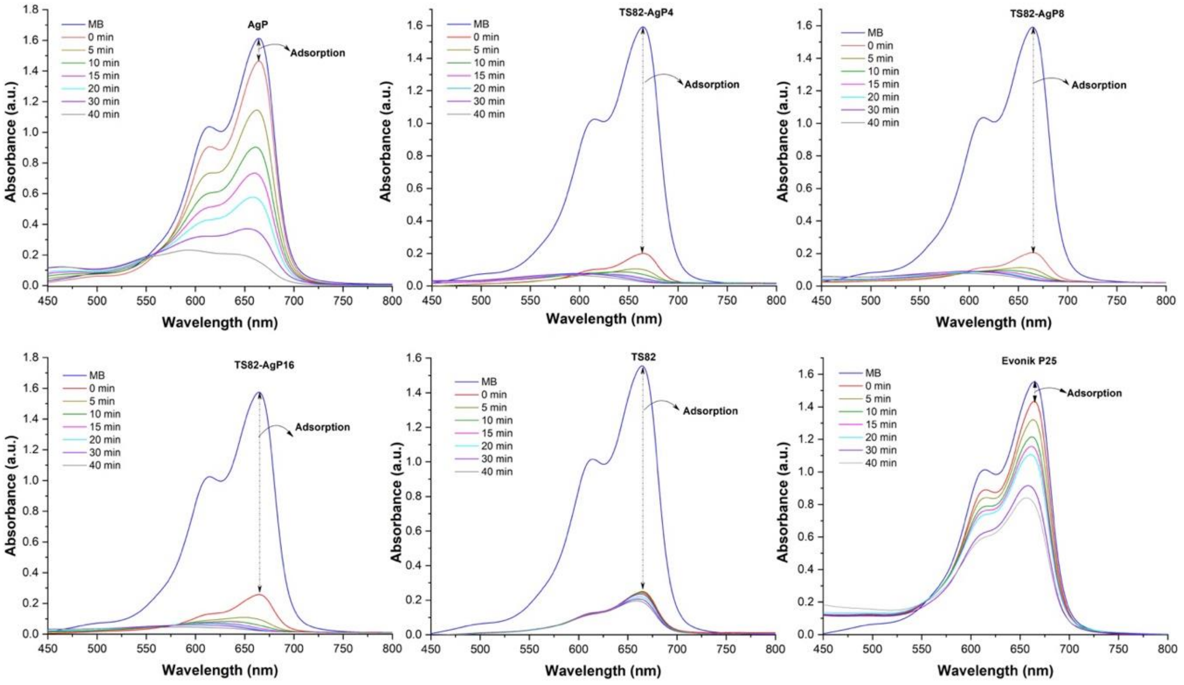

Figure S2. Evolution of the UV-Visible absorption spectra of MB dye aqueous solution (10 ppm) under solar light irradiation performed with different photocatalyst samples as indicated in the figure.

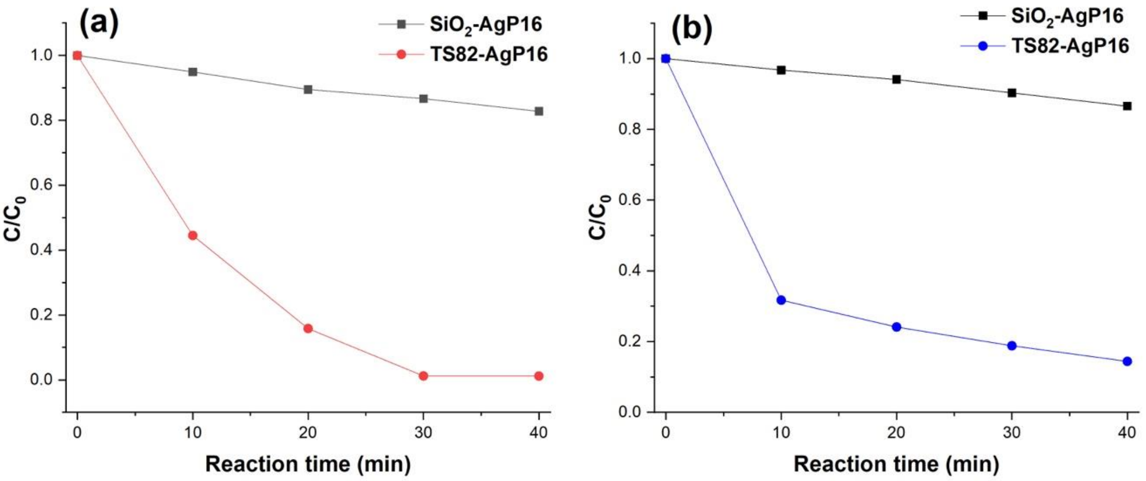

Figure S3. Plot of C/C_0 with reaction time performed with SiO_2-AgP16and TS82-AgP16 microsphere composite using (a) RhB and (b) MB dye

Table S1. Photocatalytic reaction rate constant of the corresponding samples.

Photocatalysts	RhB	MB
	[a]K (10^{-2})	[a]K (10^{-2})
TS82-AgP16	12.6	4.84
SiO_2-AgP16	0.47	0.35
